# A survey of impulse control disorders in Parkinson’s disease patients in Shanghai area and literature review

**DOI:** 10.1186/s40035-016-0051-7

**Published:** 2016-02-17

**Authors:** Xue-Ping Wang, Ming Wei, Qin Xiao

**Affiliations:** Department of Neurology and Institute of Neurology, Ruijin Hospital affiliated to Shanghai Jiao Tong University School of Medicine, Shanghai, China

**Keywords:** Parkinson’s disease, ICD, Dopamine replacement therapy

## Abstract

**Background:**

Levodopa and dopamine agonists are the main treatments for Parkinson’s disease (PD) in recent years. Increased drug dosages are linked to some severe side effects, one of which is impulse control disorders (ICD). Many studies have reported the related risk factors of ICDs, such as dopamine agonist, male sex, younger age, earlier age of onset and so on. This study aims to investigate the incidence of ICD in Chinese PD patients from Shanghai area, explore the association of ICD with dopamine replacement therapy (DRT).

**Methods:**

Two hundred seventeen PD patients were consecutively recruited from the Movement Disorder Clinic of Ruijin Hospital from March to October 2013. Minnesota Impulsive Disorders Interview was used to assess the PD patients. PD patients with possible ICD would undergo a further interview by a movement disorder specialist to confirm the diagnosis. Clinical information was also collected.

**Results:**

Nine PD patients (4.15 %) showed ICD behaviors as follows: hypersexuality (4, 1.84 %), pathological gambling (3, 1.38 %), binge eating (1, 0.46 %), compulsive shopping (1, 0.46 %). Compared with the non-ICD PD group, ICD PD group took more dopamine agonists (LED 119.4 ± 86.4 mg/d vs 60.5 ± 80.5 mg/d, *P* = 0.019), had higher total levodopa equivalent dosage (TLED 912.81 ± 878.73 mg/d vs 503.78 ± 359.14 mg/d, *P* = 0.031), and had higher H&Y stage (2.33 ± 0.87 vs 1.41 ± 0.52, *p* = 0.013). However, logistic regression analysis didn’t reveal the above factors as independent risk factors of ICD behaviors in our study.

**Conclusion:**

The incidence of ICDs behaviors in PD patients in our study is much lower than in western countries. ICD-PD group took higher dopamine agonists and higher total levodopa equivalent dosage, even though logistic regression analysis didn’t reveal them as independent risk factors.

## Background

Parkinson’s disease is associated with progressive degeneration of the nigrostriatal pathway that often impairs motor skills (resting tremor, rigidity, bradykinesia and postural instability). Dopaminergic replacement therapy (DRT), including levodopa and dopamine agonists (DA agonists), relieves the motor symptoms and improves quality of life. However, a series of motor complications, such as dyskinesia and wearing off, appear with the increased dosage of levodopa and DA agonists [1, 2]. In recent years, PD patients have been evidenced having increased risk of developing impulse control disorders (ICDs) mainly because of DRT medication. ICDs have four major symptoms, pathological gambling (PG), hypersexuality (HS), compulsive shopping (CS) and binge-eating (BE) disorder. Excessive dopaminergic medication usage, punding and aimless walkabout are also considered as impusive/compulsive behaviors (ICB) [3, 4].

Lots of studies were conducted to explore the prevalence and associated risk factors of ICDs. The most well-known was DOMINION cross-sectional study (*N* = 3090 patients) conducted by multi-centers in North America. In this study, ICD prevalence was 13.6 % (PG 5.0 %, HS 3.5 %, CS 5.7 %, and BE 4.3 %). A wide spectrum of prevalence, ranging from 3.53 % to 34.8 %, has been reported from different studies [4–12], with higher prevalence in Western populations than in Asian generally. Different variables associated with ICDs have been revealed, including DA agonist [4–6, 8, 10, 11], male sex (mainly in HS and PG) [4, 6–8, 12], younger age [6–9], earlier age of onset [9], prior personal or family history of alcohol addiction or gambling problems etc. [6, 10, 13]. But, DA agonist was the most consistent risk factor in those studies. In DOMINION study, DA agonist treatment increased about 2- to 3.5-fold odds of having an ICD, suggesting that there existed a close relationship between DA agonists and ICDs [6]. Therefore we tried to investigate the prevalence of ICDs among Chinese PD patients from Shanghai area and explore the associated risk factors, especially DA agonist medication. The underlying reasons for different prevalence between Western population and Asians were also discussed by reviewing literature.

## Methods

Two hundred seventeen idiopathic PD patients, based on UK Brain Bank clinical diagnostic criteria [14], were recruited from the Movement Disorder Clinic at the Department of Neurology of Ruijin Hospital from March to October in 2013. Exclusion criteria included atypical parkinsonism, secondary parkinsonism, and cognitive abnormality that might have problem in understanding and giving feedback of questionnaire. The study was approved by ethics committee of Ruijin Hospital.

The modified version of Minnesota Impulsive Disorders Interview (Chinese version) was used to assess gambling, compulsive shopping, hypersexuality, binge eating, and punding. The questionnaire was performed by two graduates who had been well trained and the screened positive patients were interviewed by a movement disorder specialist, and a final diagnosis was made according to the diagnostic criteria listed in Voon et al’s paper [13].

### Statistics

Statistical analysis was performed using SPSS 18.0, and *P* < 0.05 was considered to be significant. Independent-sample t-tests, non-parametric test and Fisher’s exact test were used to compare age, disease duration, Hohn-Yahr stage, gender, and dosage of anti-parkinsonian drugs between PD patients with and without ICD behaviors. Logistic regression was used to investigate the correlation among the potential risk factors for ICD behaviors.

## Results

### Demographic characteristics

The clinical characteristics are listed in Table 1. There were 120 male (55.3 %), and 97 female (44.7 %). The mean age was 67.15 ± 0.61 years old (range: 40–85) and mean disease duration was 5.78 ± 4.32 years (range:1–23). All PD patients were taking anti-parkinsonian drugs. Totally, 193(88.9 %) patients were taking levodopa with an average daily dose 494.94 ± 348.03 mg/d (100-2825 mg/d), 101(46.5 %) patients were taking DA agonists, with a dopamine agonists levodopa equivalent dosage (DA-LED) 135.15 ± 66.8 mg in average per day (ranging from 50 to 300 mg) and total levodopa equivalent dosage (TLED) 548.53 ± 382.12 mg/d (ranging from 50 to 3000 mg) (Table 1).

**Table 1:** The demographic and clinical characteristics of PD patients

Item	mean ± SD	Range
Number of cases	217	
Male, n(%)	120 (55.3 %)	
Age, year	67.15 ± 0.61	40-85
Disease duration, year	5.78 ± 4.32	1-23
H&Y stage	1.43 ± 0.55	1-4
Use of levodopa, n(%)	193 (88.9 %)	
Use of DA agonists, n(%)	101 (46.5 %)	
Levodopa, (mg/d)	494.94 ± 348.03	100-2825
DA agonist LED, (mg/d)	135.15 ± 66.8	50-300
TLED, (mg/d)	548.53 ± 382.12	50-3000

Note:DA agonist-LED (DA-LED, mg/d) = piribedil (mg/d) × 1 + pramipexole (mg/d) × 100. Total LED (TLED, mg/d) = regular levodopa dose (mg/d) × 1 + levodopa CR dose (mg/d) × 0.75 + DA-LED, and plus [regular levodopa dose (mg/d) + CR levodopa dose(mg/d) × 0.75] × 0.33 if taking COMT-I [24]

### Point prevalence and clinical features of ICD

Among 217 PD patients interviewed, 9 (4.15 %) patients fulfilled criteria for ICDs and their behaviors and medications were listed in Table 2. Seven patients among ICD group took both DA agonists and levodopa, two patients took levodopa only. Overall, there was 1(0.46 %) patient had CS, 1(0.46 %) patient had BE, 3(1.38 %) patients had PG, 4(1.84 %) PD patients were diagnosed with HS. All those patients showed ICD symptoms after DRT therapy.

**Table 2:** ICD behaviors in patients with Parkinson’s disease

case	gender	age	PD duration	H&Y stage	concurrent medication	DA-LED	TLED	ICD behaviors
1	M	75	6	3	L-DA+ pra	200	1100	PG
2	M	67	4	2	L-DA	0	300	PG
3	M	69	3	2	L-DA+ pra	150	450	HS
4	F	62	5	1	L-DA+ pra	150	825	CS
5	M	49	8	3	L-DA+ pra	225	3000	HS
6	M	50	5	3	L-DA+ pra	200	500	HS
7	F	73	10	3	L-DA	0	600	BE
8	F	51	4	2	L-DA + pir	100	300	PG
9	M	68	10	3	L-DA + pir + ent	50	1014.25	HS

Note: *pra* = pramipexole; *pir* = piribedil; *ent* = entacapone

*PG* = pathological gambling; *HS* = hypersexuality; *CS* = compulsive buying; *BE* = binge-eating disorder

Clinical characteristics of PD with ICD group and non-ICD PD group were listed in Table 3. There was no significant difference of age, gender, and disease duration between these two groups. But, for H-Y stage, ICD-PD group had more severe disease condition than non-ICD PD group (2.33 ± 0.87 vs1.41 ± 0.52, *p* = 0.013). In ICD group, 7 (77.8 %) out of 9 patients took DA agonists, the DA-LED was 119.4 ± 86.4 mg/d, 94 (45.2 %) of non-ICD patients took DA agonists (DA-LED 60.5 ± 80.5 mg/d). The dosage of DA agonists in ICD group was much higher than non-ICD patients (*P* = 0.019). Patients in ICD group also took more levodopa (791.67 ± 802.73 mg/d) than those in non-ICD group (425.0 ± 327.26 mg/d), although this difference didn’t reach the significant level (*P* = 0.066). TLED was also calculated, ICD group took 912.81 ± 878.73 mg/d TLED, which was much higher than that of non-ICD group (503.78 ± 359.13 mg/d) (*P* = 0.031). Variables entered into the multiple logistic regression analysis were: gender, age, disease duration, use of agonists, dosage of levodopa, DA-LED, TLED and H-Y stage. Our results didn’t reveal any independent risk factors for ICD behaviors (data was not shown).

**Table 3:** Comparison between patients with and without ICD behaviors (mean ± SD, n, %, p)

	Non-ICD	ICD	*P* value
Number of case	208	9	
Age, year	67.25 ± 8.82	63.67 ± 10.55	0. 469
Male, n(%)	114 (54.8 %)	6 (66.7 %)	0.521
Disease duration, year	5.76 ± 4.38	6.44 ± 3.17	0.33
Dose of levodopa, (mg/d)	425.0 ± 327.26	791.67 ± 802.73	0.066
DA - LED, (mg/d)	60.5 ± 80.5	119.4 ± 86.4	0.019*
TLED, (mg/d)	503.78 ± 359.13	912.81 ± 878.73	0.031*
H&Y stage	1.41 ± 0.52	2.33 ± 0.87	0.013*
Use of agonists, n(%)	94(45.2 %)	7 (77.8 %)	0.055

*p < 0.05 ICD vs Non-ICD group

### Comparison with Previous studies

A wide range of ICDs prevalence and variable risk factors of ICDs from different studies have been reported (Table 4). Generally, prevalence of ICDs in Asian countries (3.53 %-5.9 %) [4, 10, 11] was lower than Western countries (8.1 %-34.8 %) [5–8], except studies from Japan with 12.9 % of ICD [9] and Malaysia with 15.4 % of screen positive ICD [12]. Lower DA agonist medication, ethnic differences, social factors, and culture differences have been considered as potential factors influencing different prevalence [4, 10, 11]. But we found that screening instruments might be another influencing factor. As listed in Table 4, a variety of screen questionaires, such as Questionnaire for Impulsive Compulsive Disorders in Parkinson’s Disease (QUIP), Minnesota Impulsive Disorders Interview (MIDI) and a list of screen/diagnostic tests for PG, HS and CS, were used in these studies. Studies using QUIP as screen instrument reported high rate of screen positive ICD patients, 28 % in Japan [9], 15.4 % in Malaysia [12], 34.8 % in Finland [7] and 18.4 % in Brazil [5]. In this Japan study, the actual prevalence of ICDs assessed by various diagnostic criteria for each ICD turned out to be 12.9 %. Thus, QUIP might be an optimal instrument for screening ICDs because of its high sensitivity, but further diagnostic criteria is needed for confirmative diagnosis.

**Table 4:** Comparison between previous studies and present study

Study population	Sample Size	Screen instruments	Frequency of compulsive behaviors	Risk factors	Dosage of drugs (ICD vs non ICD mg/d, mean)	Reference
Taiwan	268	DSM-IV-TR criteria for PG and BE, McElroy’s criteria for CS Voon’s criteria (2006) for HS	ICD 4.48 % PG 1.49 %, HS 2.99 % CS 0 %, BE 0.37 %	Dopamine agonist TLED Male gender	TLED (ICD group): 741.94 DA-LED (ICD group): 105	4
Brazil	152	QUIP	QUIP results: ICD 18.4 % PG 1.3 %, HS 11.8 % CS 10.5 %, BE 7.9 %	Smoking Use of pramipexole	TLED: 732.8 vs 644.4 Pramipexole: 2.9 vs 0.85 (equivalent to 290 vs 85)	5
North America	3090	Massachusetts Gambling Screen MIDI DSM-IV-TR	ICD 13.6 % PG 5 %, HS 3.5 % CS 5.7 %, BE 4.3 %	DA combination of DA & Levodopa younger age, male sex current smoking being unmarried living in the United States family history of gambling	DA-LED: 200 ~ 450 vs 150 ~ 400 (listed as interquartile in patients taking DA) Levodopa: 621 vs 461 (in patients taking levodopa only)	6
Finland	575	SOGS QUIP	QUIP results: ICD 34.8 % PG 8.8 %, HS 22.8 % CS 10.1 %, BE 11.8 %	Depression, Male sex Younger age Age of disease onset Current alcohol use	NA	7
Italy	805	QUIP for screen Other criteria for confirmation	ICD 8.1 % PG 3.2 %, HS 3.0 % CS 1.0 %, BE 2.4 %	dopamine agonist Male sex, levodopa younger age, Amantadine	NA	8
Japan	118	QUIP for screen Other criteria for confirmation	QUIP results vs Actual prevalence ICD 28 % vs 12.9 % PG 14 % vs 6.5 % HS 14 % vs 3.2 % CS 10.8 % vs 3.2 % BE 10.8 % vs 3.2 %	younger age longer disease duration TLEDD, Levodopa Earlier age of onset	ICB vs non-ICB Levodopa: 676 vs 520 DA-LED: 139 vs 71.6	9
China	312	SOGS, DSM-IV Lejoyeux’s Compulsive Shopping questionnaire; specially designed hypersexuality questionnaire;	ICD 3.53 % PG 0.32 %, HS 1.92 % CS 0.32 %, BE 0.32 %	Dopamine agonist Alcohol use	Levodopa: 345.5 vs 357.01 DA-LED: 142.37 vs 35.45	10
South Korea	1167	MIDI	ICD 5.9 % PG 1.3 %, HS 2.8 % CS 2.5 %, BE 3.4 %	Dopamine agonist Levodopa	Levodopa: 656 vs 544 DA-LED: 145 vs 99	11
Malaysian	200	QUIP	QUIP results based on patient report ICD 15.4 % PG 2.6 %, HS 8.2 % CS 3.6 %, BE 8.7 %	Male gender Longer PD duration	PD patients: TLED: 527.7 DA-LED: 73.7	12
Present study	217	MIDI Voon’s criteria (2007) for each ICD	ICD 4.15 % PG 1.38 %, HS 1.84 % CS 0.46 %, BE 0.46 %	None	Levodopa: 791.67 vs 425.0 DA-LED: 119.4 vs 60.5	--

*SOGS*: modified South Oaks Gambling Screen. *QUIP*: Questionnaire for Impulsive Compulsive Disorders in Parkinson’s Disease

*MIDI*: Minnesota Impulsive Disorders Interview. *ICB*: impulsive compulsive behavior

*DA-LED*: Dopamine agonist levodopa equivalent dosage. *TLED*: Total levodopa equivalent dosage

## Discussion

Previous reports estimated the frequency of ICDs in PD patients ranged from 3.53 % to 34.8 % [4–12]. Two reports from Xuanwu Hospital Beijing China and ChangGung Memorial Hospital Taiwan reported the rates of ICDs in PD patients are 3.53 % and 4.48 % respectively [4, 10]. ICD frequency in our study was estimated at 4.15 %. These studies carried out among Chinese PD patients got similar results.

By comparison with Western countries reports, results from Asian studies showed a relatively low incidence of ICD except studies from Japan with 12.9 % of ICD [9] and Malaysia with 15.4 % of ICD [12]. Point prevalence of ICDs from China, Taiwan, South Korea and present study showed a range from 3.53 % to 5.9 % [4, 10, 11], while data from North America, Brazil, Italy, Finland showed higher prevalence 8.1 % ~ 34.8 % [5–8]. Note that studies from Malaysia, Brazil and Finland reported high frequency of ICDs only by QUIP screen without further confirmative diagnosis. Besides that, a variety of reasons have been considered for different prevalence. Lower dosage of DA agonists because of medication practice, cost burden, limited availability and health insurance [10] has been suggested as a potential factor affecting different prevalence. Other factors, such as ethnic differences, social factors, study design, culture difference, were also considered [4–12]. Genetic factors have also been revealed. Polymorphism of dopamine receptor D3 and D2, serotonin 2A receptor gene (HTR2A) have been linked with ICDs [15–17].

However, here we want to emphasize screen instruments as a potential reason for different prevalence. Study from Japan reported 28 % ICDs by QUIP screen and 12.9 % actual prevalence by other diagnostic criteria [9], indicating QUIP is a screening test with high sensitivity but relatively low specificity. QUIP is a comprehensive screening test and can evaluate a wide range of impulsive compulsive behaviors [18]. In our study, we used MIDI for screen test. MIDI and QUIP are different in design. MIDI has 5 modules with 1 module for each ICD. Each module has one Yes/No question for preliminary screen, and the Yes/No answer largely depends on the patients or informants’ rough judgement. In QUIP test with session 1 as an example, for each ICD there are 5 specific questions from different perspectives collecting patients’ feelings and experiences, and patients or informants can make a fine judgement based on those 5 questions. Thus, MIDI is a quick and convenient battery for clinical work, while QUIP is more comprehensive and sensitive for both research and clinical work. But, to get a final diagnosis, other diagnostic criteria were needed. In our study, diagnostic criteria listed by Voon et.al [13] were adopted.

With regard to potential risk factors for ICDs identified in previous analyses, such as younger age, male sex, earlier age of disease onset, were not revealed as independent risk factors for development of ICDs in our study. Many researchers have confirmed that DA agonist was a main risk factor for ICDs [4–6, 8–11, 19]. However, logistic regression analysis didn’t reveal it as an independent risk factor for ICD in our study. Failing to identify DA agonist as a risk factor might due to several reasons. First, the main reason might be lower average daily dosage of DA agonists (ICD vs non-ICD: 119.4 ± 86.4 vs 60.5 ± 80.5 mg/d) taken by our patients compared with other studies listed in Table 4. Study from Malaysia didn’t find correlation between DA agonist and ICD either. There were less than 50 % PD patients taking DA agonist and the average daily dosage of DA agonist was 73.7 ± 84.3 mg in that study [12]. Therefore, high dosage of DA agonist places patients in a higher risk condition. Lower dosage might cover the potential relationship between drugs and development of ICD in this study. Second, it might be due to sample and area limitation in our study. PD patients enrolled in our study were mainly from Shanghai. Larger sample and a multi-center based study would be a better option to explore risk factors in Chinese population.

Although failing to identify DA agonist as a risk factor, we found a higher percentage of ICDs patients took DA agonists and a higher average daily DA agonist dosage than that of non-ICD patients, which also indicates DA agonist might be a potential risk factor for those PD-ICD patients. One patient with HS behavior in our study got recovered by discontinuation of pramipexole. Previous study showed successful management of the PD patient with ICD by reduction or discontinuation of DA agonist therapy [19–23]. Seven of 18 PD patients with an ICD had resolution of the behavior by discontinuation or dosage reduction of a DA agonist [22]. A 43 months follow–up study showed that nearly 73 % of PD patients with ICD behaviors were completely recovered after reducing dosage of DA drugs [23]. At present, there were very limited data to support an effective medicine for treatment of ICD behaviors in PD. Reduction or discontinuation of DA agonist is generally accepted as the first line management strategy of ICDs [23], especially for those PD patients had ICD behaviors after DRT therapy.

## Conclusions

In conclusion, the incidence of ICDs behaviors in PD patients in our study is much lower than in western countries. Routine screening of ICDs in PD patients is necessary to discover PD-ICD patients and give early intervention.
